# 肺腺癌骨转移裸小鼠模型的建立及MicroCT观察

**DOI:** 10.3779/j.issn.1009-3419.2013.09.03

**Published:** 2013-09-20

**Authors:** 永奇 崔, 沁 耿, 爱琴 顾, 淼鑫 朱, 韩卫 孔, 磊 孙, 蕾 刘, 明霞 闫, 明 姚

**Affiliations:** 1 200032 上海，上海交通大学医学院附属仁济医院上海市肿瘤研究所癌基因及相关基因国家重点实验室 State Key Laboratory of Oncogenes and Related Genes, Shanghai Cancer Institute, Renji Hospital, Shanghai Jiaotong University School of Medicine, Shanghai 200032, China; 2 200030上海，上海交通大学附属胸科医院 Toracic Hospital Afliated to Shanghai Jiaotong University, Shanghai 200030, China

**Keywords:** 肺腺癌, 骨转移, 裸小鼠模型, MicroCT, Lung adenocarcinoma, Bone metastasis, Nude mice model, MicroCT

## Abstract

**背景与目的:**

骨转移占晚期肺癌的50%-70%。本研究以体外侵袭、迁移能力不同的肺腺癌细胞系A549、H1299、SPC-A-1、XL-2为基础建立肺腺癌骨转移裸小鼠模型，MicroCT观察骨转移情况。

**方法:**

将50只6 w-8 w龄裸小鼠随机平均分为5组，4个实验组左心室分别注射相应四种细胞悬液（0.2 mL/只）；对照组左心室注射等量生理盐水。注射后第二周起定期对各组小鼠进行MicroCT扫描，当小鼠明显消瘦时此组观察结束，结束前行骨组织病理学检查；对各实验组出现的骨转移部位按中轴骨和四肢骨归类，比较这两种部位之间的转移率；根据各组出现骨转移所用平均时间、骨转移率，对各细胞系骨转移能力进行统计分析。

**结果:**

经MicroCT、病理学检查确定，各实验组出现不同骨转移率，对照组小鼠无骨转移现象；各实验组中轴骨转移率均明显高于四肢骨，这与临床上肺癌骨转移规律一致，模型建立成功。各实验组间发生骨转移的小鼠数目及出现转移所用平均时间无明显差异。

**结论:**

MicroCT能清晰地检测到骨质破坏，利于骨转移情况的判断；我们成功建立了肺腺癌骨转移模型，为以后探索出新的肺腺癌乃至肺癌骨转移临床预防和治疗方案提供基础；4种肺腺癌细胞系体外侵袭、迁移能力强弱不等，但体内骨转移能力没有明显差异，其原因还有待进一步的探索。

## Abstract (en)

**Background and objective:**

50%-70% of patients with advanced lung cancer will develop bone metastases. The aim of this study is to establish the nude mice bone metastasis model of lung adenocarcinoma using A549, H1299, SPC-A-1 and XL-2, all of which own different invasion and migration abilities *in vitro* and supervise the bone metastases by MicroCT.

**Methods:**

fifty BALB/C-nu/nu nude mice were grouped into five groups on average randomly. Cells of the four cell lines were injected into the left cardiac ventricle of mice in the four experimental groups (0.2 mL/mouse) respectively; meanwhile, mice in the control group were injected with normal saline (0.2 mL/mouse) in the same manner. Periodical radiological examination was carried out to supervise the variation of the mice since the second week after injection. When mice in each group became thin obviously, end the experiment of this group. Before the end, pathological sections of bone tissues were made. We classified the bone metastatic sites into axial skeleton and limb bone, in order to compare the metastatic rates of these two different parts. The bone metastatic abilities of the four cell lines was statistically analyzed by comparing the average time cost in the appearance of bone metastases and the percentage of bone metastases among the experimental groups.

**Results:**

Different metastatic sites which had been identified both by MicroCT and pathological sections appeared in each group of the four experimental groups. By contrast, no metastasis was observed in the control group. The percentage of cancer metastasizing to axial skeleton was remarkably higher than the percentage of tumor metastasizing to the limb bone in each experimental group, which was consistent with the clinical regularity and characteristics of skeletal metastases with lung cancer. Thus, the model has been established triumphantly. However, there were no statistical differences in the average time consumed and skeletal metastatic rate among the four experimental groups.

**Conclusion:**

The disruption in the bone can be clearly detected by MicroCT, which is benefit to supervise the osseous metastasis. We successfully developed the nude mice bone metastasis model of lung adenocarcinoma, which will pave the way for exploring novel prevention and therapy strategies clinically. The four cell lines varied in invasion and migration abilities *in vitro*, but there was no statistical difference in the metastatic ability *in vivo*, and the reason need to be explored further in future.

肺癌是全球范围内发病率和致死率最高的恶性疾病之一，大约90%的肺癌患者死于转移^[1, 2]^，而骨转移占晚期肺癌的50%-70%^[3]^，这将进一步引起高血钙症、神经压迫、残疾、病理性骨折等症状，导致患者生活质量的迅速下降^[4]^。但是，目前在治疗肺癌骨转移方面我们取得的成果还很少，尚且没有一种完全有效的方法来预防和治疗骨转移。由于肺癌骨转移灶的形成是一个多因素、多步骤的复杂的生物学过程^[5]^，因此，一个合适的可以用来了解和探讨肺癌骨转移机制的小动物模型的建立就显得尤为重要，这对于以后探索出新的肺癌骨转移临床预防和治疗方案具有深远的意义。

由于在肺癌的几种病理类型中腺癌的骨转移率最高^[6]^，因此本实验我们以几种肺腺癌细胞系为基础进行肺癌骨转移裸小鼠模型的建立，希望能为以后进行肺癌骨转移机制的研究提供新的线索。

## 1 材料与方法

### 1.1 材料

#### 1.1.1 细胞系

本实验使用了四种肺腺癌细胞系：A549、NCI-H1299（以下简称H1299）、SPC-A-1、XL-2。其中A549、H1299由ATCC提供，SPC-A-1由上海市胸科医院提供，XL-2^[7]^是我们实验室从发生全身转移的肺腺癌病人的腹水中分离并建系。

#### 1.1.2 实验动物

SPF级BLAB/c-nu/nu裸小鼠50只，雌雄兼用，6 w-8 w鼠龄，体重20 g-22 g，由上海市肿瘤研究所提供，实验动物生产许可证号为SCXK (沪) 2012-0001；所有小鼠均在无特殊病原体(specific pathogen free, SPF)级环境中饲养，饲料和水自由摄入，使用许可证号为SYXK (沪) 2012-0001。

#### 1.1.3 主要试剂、耗材

胎牛血清FBS（Biowest, South America Origin），4%多聚甲醛固定液和EDTA脱钙液(上海威奥生物科技有限公司）、Transwell小室（8 μm pore size, Corning, USA）、Matrigel Matrix（BD Bioscience, USA）

#### 1.1.4 仪器设备

微计算机断层扫描技术（micro computed tomography, MicroCT）（skyscan1076，比利时）、全自动病理分析系统（Leica，德国）。

### 1.2 方法

#### 1.2.1 细胞培养

A549、H1299、SPC-A-1、XL-2四种细胞系培养条件为DMEM培养基，内含10%胎牛血清和青霉素（100 U/mL）及链霉素（100 mg/mL），细胞培养条件为37 ℃、5%CO_2_。

#### 1.2.2 细胞增殖能力

四种细胞均取2, 000个加入到96孔板各孔内，每天各种细胞均取3个孔，每孔加CCK8（Dojindo, Japan）、DMEM混合液110 μL，2 h后检测450 nm波长处吸光度以计算每孔内活细胞的数目。实验独立重复3次。

#### 1.2.3 细胞体外侵袭、迁移能力

将冻存于-20 ℃ Matrigel胶放于4 ℃过夜，使其由凝固状态变为液态；用DMEM将其稀释到1 mg/mL，并迅速加入到Transwell上室内膜上、铺平，每个小室40 μL，37 ℃孵育2 h。用DMEM悬浮细胞，浓度调整至1×10^5^/0.2 mL，接种于各上室，在各下室加入800 μL含10%FBS的DMEM培养基，37 ℃培养箱中孵育，18 h后取出小室，弃去培养基，用棉签拭去内表面未侵袭细胞；将各个Transwell放入100%甲醇中室温固定30 min，弃去各个上室内甲醇后，再将其放入0.1%结晶紫中染色30 min，然后PBS洗2遍，棉签将小室内表面擦拭干净，倒置显微镜下拍照，计数迁移到小室外表面的细胞数目，取平均值，实验重复3次。细胞迁移实验与侵袭实验不同之处：上室不需加入DMEM稀释的Matrigel胶，细胞浓度为5×10^4^个/0.2 mL，孵育时间为12 h左右。

#### 1.2.4 模型建立

##### 1.2.4.1 实验分组

将50只裸小鼠随机分为5组，每组10只，其中一组为对照组，每只左心室注射0.2 mL生理盐水，另外四组为实验组，每只左心室注射0.2 mL相应四种肺腺癌细胞悬液，分别命名为A549实验组、H1299实验组、SPC-A-1实验组、XL-2实验组。

##### 1.2.4.2 左心室注射

将以上四种细胞于细胞对数生长期消化下来，加入不含血清的DMEM培养基，调整细胞浓度为1×10^6^/0.2 mL，将小鼠仰卧固定在固定板上，用1%戊巴比妥钠按照每克小鼠体重0.01 mL的剂量进行腹腔注射麻醉，酒精棉球常规消毒小鼠前胸壁，1 mL胰岛素注射器（29 G, BD Bioscience）抽吸0.2 mL细胞悬液于胸骨左旁3 mm第二肋间、与胸廓成45度夹角，对准正中进针，一般进针6 mm，当针尖头有自动持续的鲜红色动脉血不断搏动地进入针芯即表明真尖已进入左心室。对照组小鼠用同样的方法进行注射，每只仅注射0.2 mL生理盐水。术后继续在SPF环境中饲养，并密切观察小鼠状态。

#### 1.2.5 MicroCT扫描

观察小鼠状态，自左心室注射术后第2周起定期对各组小鼠运用MicroCT进行扫描（条件为40 KV，250 mA，分辨率35 μm），以确定是否已发生肺癌骨转移以及骨转移部位，并进行拍照，三维重建。

#### 1.2.6 组织病理学鉴定

以小鼠出现明显消瘦为这组实验结束点。选取实验组及对照组小鼠中轴骨（如：肋骨、脊柱、髂骨、肩胛骨）、四肢长骨等部位骨组织，将其截成0.5 cm×0.3 cm×0.2 cm大小骨片，生理盐水清洗后立即投入冷冻的4%多聚甲醛固定液中，4 ℃固定12 h-24 h；固定结束后骨片在0.2 mol磷酸缓冲液中充分漂洗。漂洗结束后投入EDTA脱钙液中，室温下脱钙，脱钙液每4天-5天更换一次新液，经过3次更换新液后，此后每天更换新液，直至骨片完全脱钙为止，每日检查脱钙情况，以大头针能刺进骨密质为完成脱钙标准。脱钙结束后，骨片用蒸馏水冲洗20 min，然后进行常规脱水处理、石蜡包埋、切片（切片厚度4.5 μm），行苏木素-伊红染色。

#### 1.2.7 统计学分析

数据采用SPSS 19.0统计软件进行统计分析。计量资料用Mean±SD表示，组间比较采用卡方检验或单因素方差分析，*P* < 0.05为具有统计学差异。

## 2 结果

### 2.1 四种细胞体外增殖能力分析

如图 1所示。

**1:**
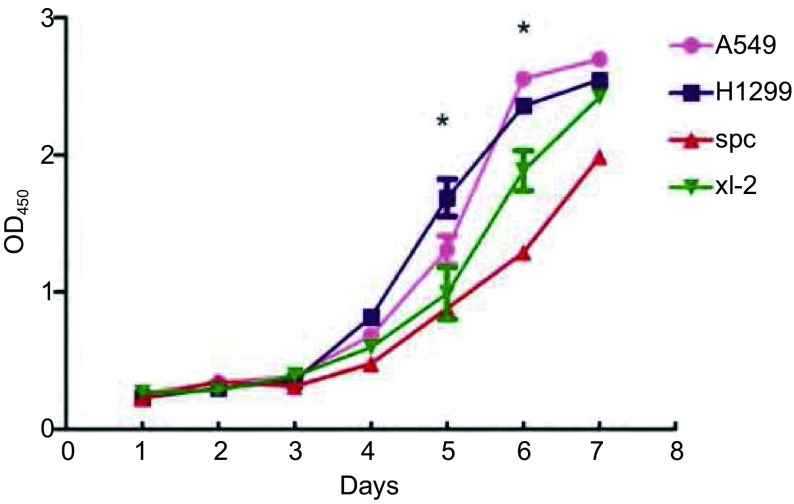
体外生长曲线检测四种细胞之间群体细胞增殖能力。^*^P < 0.05，差异具有统计学意义。 *In vitro* cell growth curve assay of four cell lines. ^*^P < 0.05, there was statistical significance among the proliferation ability of the four cell lines.

### 2.2 四种细胞之间体外侵袭、迁移能力比较

Transwell结果显示四种细胞体外无论是侵袭能力还是迁移能力均按A549、H1299、SPC-A-1、XL-2的顺序由高到低排列，具体结果如图 2所示。

**2:**
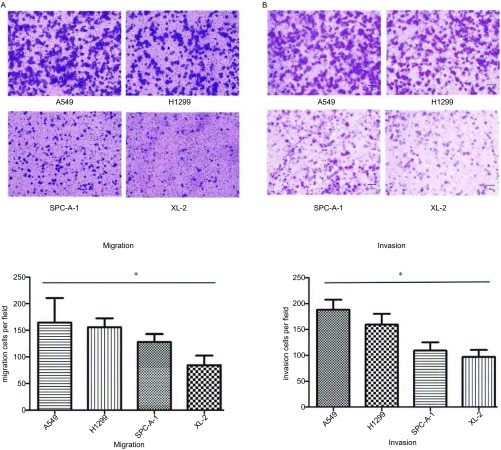
体外侵袭、迁移实验检测四种肺腺癌细胞系之间侵袭、迁移能力的差异。A：四种肺腺癌细胞之间迁移实验（×100）；B：四种肺腺癌细胞之间侵袭实验（×100）；^*^P < 0.05，四种细胞之间侵袭、迁移能力差异均有统计学意义。 *In vitro* invasion and migration assays of 4 lung adenocarcinoma lines. A: The migration ability of 4 lung adenocarcinoma lines using the migration assay (×100); B: The invasion ability of the 4 lung adenocarcinoma lines using the matrigel invasion assay (×100); ^*^P < 0.05, there was statistical significance.

### 2.3 模型建立

#### 2.3.1 模型建立情况

5组小鼠左心室注射后全部成活。至实验结束，对照组小鼠生长状况良好，无明显消瘦、活动障碍等情况；实验组小鼠每组术后均有一定概率的消瘦，脊柱弯曲、肋骨、肩胛骨、下颌骨、髂骨、四肢长骨等不同部位的骨转移现象发生，每组小鼠骨转移率及具体骨转移部位见表 1、表 2。

**1:** 四个实验组每组小鼠出现骨转移所用平均时间及每组骨转移概率 The average time cost and the bone metastatic rate of each experimental group

Cell lines	Average time (d)	Bone metastases^∆^
A549	33±1^*^	60% (6/10^*^^*^)
H1299	57±4	40% (4/10)
SPC-A-1	32±3	50% (5/10)
XL-2	15±2	40% (4/10)
^*^: The average time from the left cardiac ventricle injection to the appearance of the bone metastases consumed in each group; ^**^: The number of mice with bone metastases identified by radiological examination and pathological sections/the total number of mice in each experimental group; ^∆^: There was no statistical difference in the bone metastatic rate among the experimental groups.		

**2:** 各实验组骨转移部位分析 Analysis on the bone metastatic sites of the experimental groups

Cell lines	Axial skeleton^∆^	Limb bone
A549	67% (4/6)^*^	33% (2/6)
H1299	75% (3/4)	25% (1/4)
SPC-A-1	80% (4/5)	20% (1/5)
XL-2	75% (3/4)	25% (1/4)
^*^: The number of the mice with metastases to the axial skeleton/the total number of the mice with bone metastases in each experimental group; ^∆^: There was statistical significance between the metastatic rate of axial skeleton and limb bone in each experimental group.		

#### 2.3.2 MicroCT结果

对照组小鼠MicroCT显示骨密度均匀，未见骨质缺损、骨折现象发生；实验组小鼠每组内均可见脊柱、肩胛骨、下颌骨、四肢长骨等不同部位低密度病灶，骨皮质缺损、骨组织破坏严重（图 3）。

**3:**
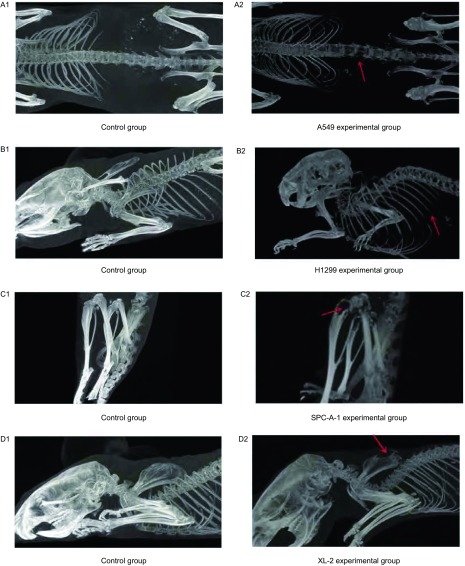
对照组及各实验组小鼠MicroCT。A1-D1：对照组小鼠正常脊柱、肋骨、胫骨、肩胛骨MicroCT结果；A2：A549实验组内出现的脊柱转移；B2：H1299实验组内出现的肋骨转移；C2：SPC-A-1实验组内出现的胫骨破坏；D2：XL-2实验组内出现的肩胛骨变化；箭头所示处为各部位的骨质破坏。 MicroCT of the mice in the assay. A1-D1: MicroCT of the mice in the control group show the normal spinal column, rib, tibia and scapula; A2: MicroCT of the mice in the A549 experimental group shows the spinal column metastasis; B2: MicroCT of the mice in the H1299 experimental group shows rib metastasis; C2: MicroCT of the mice in the SPC-A-1 experimental group indicates tibia metastasis; D2: MicroCT of the mice in the XL-2 experimental group indicates scapula metastasis; Arrows show different bone metastatic sites of lung cancer.

#### 2.3.3 组织病理学结果

对照组小鼠骨组织切片正常，未发现转移灶；每个实验组小鼠骨组织切片经HE染色发现有肩胛骨、股胫骨、肋骨、脊柱等不同部位的肿瘤转移灶：骨髓腔内成片的肿瘤细胞排列紊乱、细胞分化差、核大浓染，骨组织结构消失，如图 4所示。

**4:**
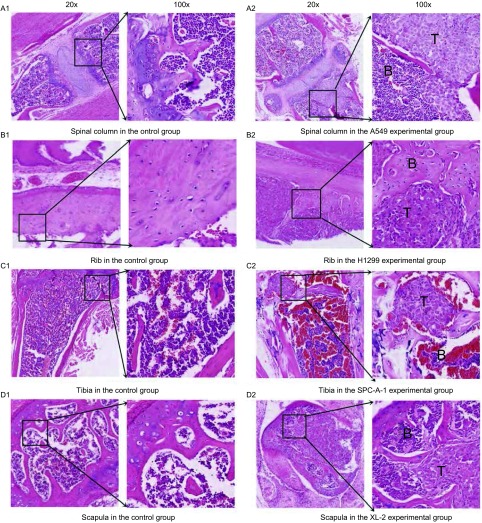
实验组及对照组小鼠骨切片HE染色。A1：对照组小鼠正常脊柱；A2：A549实验组小鼠脊柱发生肿瘤转移的情况；B1：对照组小鼠正常肋骨；B2：H1299实验组小鼠肋骨处肿瘤转移情况；C1：对照组小鼠正常胫骨；C2：SPC-A-1实验组肿瘤胫骨转移；D1：对照组小鼠正常肩胛骨；D2：XL-2实验组小鼠发生肿瘤肩胛骨转移。 HE staining was performed to confirm the bone lesion in the control group and experimental groups. A1: The normal spinal column in the control group; A2: Spinal column with tumor metastasis in the A549 experimental group; B1: Normal rib in the control group; B2: Rib with tumor metastasis in the H1299 experimental group; C1: Normal tibia in the control group; C2: Tibia with tumor metastasis in the SPC-A-1 experimental group; D1: Normal scapula in the control group; D2: Scapula with tumor metastasis in the XL-2 experimental group; T: Tumor, B: Bone.

### 2.4 骨转移分析

#### 2.4.1 四个实验组骨转移结果

体内实验每个实验组出现转移的小鼠数目、出现转移症状所用时间如表 1所示。

#### 2.4.2 转移部位分析

至实验结束A549实验组、H1299实验组、SPC-A-1实验组、XL-2实验组出现骨转移的小鼠总数依次为6只、4只、5只、4只，对每个实验组内出现的骨转移按照中轴骨、四肢长骨进行归类，具体结果如表 2。

## 3 讨论

目前对于骨转移模型建立的研究比较多的集中于乳腺癌^[8, 9]^及前列腺癌^[10]^，而对于肺癌骨转移模型建立的报道还比较有限，腺癌作为肺癌中骨转移率最高的一种病理类型，对其骨转移机制的探索越来越引起人们的重视。针对这一特点，本实验经过大量预实验的探索，最终通过左心室注射的方法获得了经影像学、组织病理均证实发生了骨转移的肺腺癌裸小鼠模型。临床上肺癌骨转移的部位通常首先累及中轴骨，其次为股骨、肱骨等四肢长骨，本实验中四个实验组内每组出现中轴骨转移的概率均明显高于四肢长骨，这与Coleman等^[11, 12]^研究的结论一致，肺腺癌骨转移裸小鼠模型建立成功。

左心室注射法是目前使用较广泛的一种肿瘤转移动物模型制作方法^[13]^，有很多优点，并且能验证“种子与土壤”学说^[14]^：肿瘤细胞充当种子，骨髓微环境充当土壤。在肿瘤细胞从原发灶脱落开始直至新的转移灶形成的过程中，这种方法建立的骨转移模型模拟的是肿瘤细胞从进入血液循环开始，随着血流到达骨的毛细血管床、穿过血管内皮间隙进入骨组织、与骨组织微环境经过一系列相互作用、在骨内增殖和导致骨质重构的过程；这与胫骨内注射法建立的乳腺癌及前列腺癌等恶性肿瘤的骨转移模型相比更接近于肿瘤转移的真实过程，因为后者模拟的只是肿瘤细胞定位于骨组织后的过程；所以，使用左心室注射的方法建立的肺癌骨转移模型形成的骨转移灶更与临床相似；并且，胫骨内接种的方法表现的骨转移主要为长骨骨干转移，这与临床上常见的椎体及骨骺端转移有所不同。

但左心室注射法由于易导致脑、肺、肾上腺皮质等其他部位和器官多发性转移的缺点^[15-16]^，以致骨转移灶尚未形成，实验动物已死于其他部位转移；再加上样本量有限等，可能导致了本实验中尽管我们选用的四种肺腺癌细胞系体外侵袭、迁移能力均按A549、H1299、SPC-A-1、XL-2的顺序由高到低排列，但体内四个实验组骨转移率的高低以及出现骨转移所用平均时间的长短却未呈这样的顺序排列。至于导致这种现象的具体原因，还有待于以后更进一步的实验探索。

与传统的放射学技术相比，MicroCT分辨率高、不存在图像重叠、伪影、比例放大等不足；与传统的组织学切片技术相比，MicroCT能够在不破坏样品的情况下对骨组织、活体小动物进行成像，还能精确进行图像三维重建，它成为我们判断骨组织破坏情况较为精准的方法之一。

总之，我们成功地建立了肺腺癌骨转移裸小鼠模型，成为研究肺腺癌乃至肺癌骨转移分子机制、探索出新的临床预防和治疗方案不可缺少的工具。
